# TMS as a pharmacodynamic indicator of cortical activity of a novel anti‐epileptic drug, XEN1101

**DOI:** 10.1002/acn3.50896

**Published:** 2019-09-30

**Authors:** Isabella Premoli, Pierre G. Rossini, Paul Y. Goldberg, Kristina Posadas, Louise Green, Noah Yogo, Simon Pimstone, Eugenio Abela, Gregory N. Beatch, Mark P. Richardson

**Affiliations:** ^1^ Department of Basic and Clinical Neuroscience Institute of Psychiatry, Psychology and Neuroscience King’s College London London UK; ^2^ Xenon Pharmaceuticals Inc. Burnaby Canada; ^3^ Clinical Research Facility King’s College Hospital London UK; ^4^ Department of General Internal Medicine University of British Columbia Vancouver Canada

## Abstract

**Objective:**

Transcranial magnetic stimulation (TMS) produces characteristic deflections in the EEG signal named TMS‐evoked EEG potentials (TEPs), which can be used to assess drug effects on cortical excitability. TMS can also be used to determine the resting motor threshold (RMT) for eliciting a minimal muscle response, as a biomarker of corticospinal excitability. XEN1101 is a novel potassium channel opener undergoing clinical development for treatment of epilepsy. We used TEPs and RMT to measure the effects of XEN1101 in the human brain, to provide evidence that XEN1101 alters cortical excitability at doses that might be used in future clinical trials.

**Methods:**

TMS measurements were incorporated in this Phase I clinical trial to evaluate the extent to which XEN1101 modulates TMS parameters of cortical and corticospinal excitability. TEPs and RMT were collected before and at 2‐, 4‐, and 6‐hours post drug intake in a double‐blind, placebo‐controlled, randomized, two‐period crossover study of 20 healthy male volunteers.

**Results:**

Consistent with previous TMS investigations of antiepileptic drugs (AEDs) targeting ion channels, the amplitude of TEPs occurring at early (15–55 msec after TMS) and at late (150–250 msec after TMS) latencies were significantly suppressed from baseline by 20 mg of XEN1101. Furthermore, the RMT showed a significant time‐dependent increase that correlated with the XEN1101 plasma concentration.

**Interpretation:**

Changes from baseline in TMS measures provided evidence that 20 mg of XEN1101 suppressed cortical and corticospinal excitability, consistent with the effects of other AEDs. These results support the implementation of TMS as a tool to inform early‐stage clinical trials.

## Introduction

Epilepsy is a very common disorder in which approximately one third of patients do not respond to available antiepileptic drugs (AEDs).^1^ There is therefore a continued need to develop more efficacious AEDs. One of the crucial challenges for early‐stage development of new AEDs is to assess whether the investigational compound can cross the blood brain barrier (BBB) and whether it is acting at the intended target. Positron emission tomography (PET) or collection of cerebrospinal fluid can provide evidence, however, they are invasive, expensive, and not easily accessible. Potentially, electro‐diagnostic markers (EDM) could provide an efficient and reliable way to demonstrate the cortical activity of AEDs.

Transcranial magnetic stimulation (TMS) is a non‐invasive brain stimulation technique.^2^ Responses resulting from motor cortex stimulation can be measured peripherally with electromyographic (EMG) recording over contralateral muscles or more directly via electroencephalography (EEG). Multiple AEDs and other drugs acting in the brain have shown effects on TMS‐EMG^3^ and TMS‐EEG^4^ measures. The TMS‐EEG response shows alternating positive and negative peaks at characteristic latencies, called TMS‐evoked EEG potentials (TEPs).^5^ TEPs represent the summation of TMS‐induced excitatory and inhibitory neuronal processes.^6^ Although the complete physiological mechanism underlying TEPs is still unresolved, a growing body of evidence indicates that TMS‐EDM can reliably detect drug‐induced changes in cortical excitability. Thus, TMS‐EDM has significant potential to serve as a reliable and efficient biomarker to demonstrate in early‐stage clinical trials that a drug can cross the BBB and engage its relevant target.

Here, we provide proof‐of‐principle for this approach in a phase I clinical trial of XEN1101, an AED in early stage clinical development. XEN1101 is a novel potassium channel opener with the same mechanism of action as retigabine. We hypothesized that XEN1101 would show TMS‐EDM effects, modulating cortical excitability consistent with previous TMS studies of AEDs.^7^, ^8^, ^9^ As predicted, we found that XEN1101 had strong effects on RMT and TEPs, which suggests that it reduces cortical excitability. This study provides evidence for the feasibility and utility of TMS for the assessment of AED target engagement and pharmacodynamic effects early during drug development.

## Method

### Drug information

XEN1101 is a novel positive allosteric modulator (“opener”) of the potassium channel KCNQ2/3 (Kv7.2/7.3) currently being developed by Xenon Pharmaceuticals Inc. for the treatment of focal epilepsy. Potentiating the open state of KCNQ2/3 channels favors a hyperpolarized resting state.^10^ This mechanism has been clinically proven effective for focal onset seizures using the KCNQ2/3 opener, retigabine.^11^, ^12^, ^13^

### Phase I clinical study

The experimental protocol was approved by the MHRA, local ethics committees, and all participants gave written informed consent (ClinicalTrials.gov Identifier: NCT03468725).

**Figure 1:**
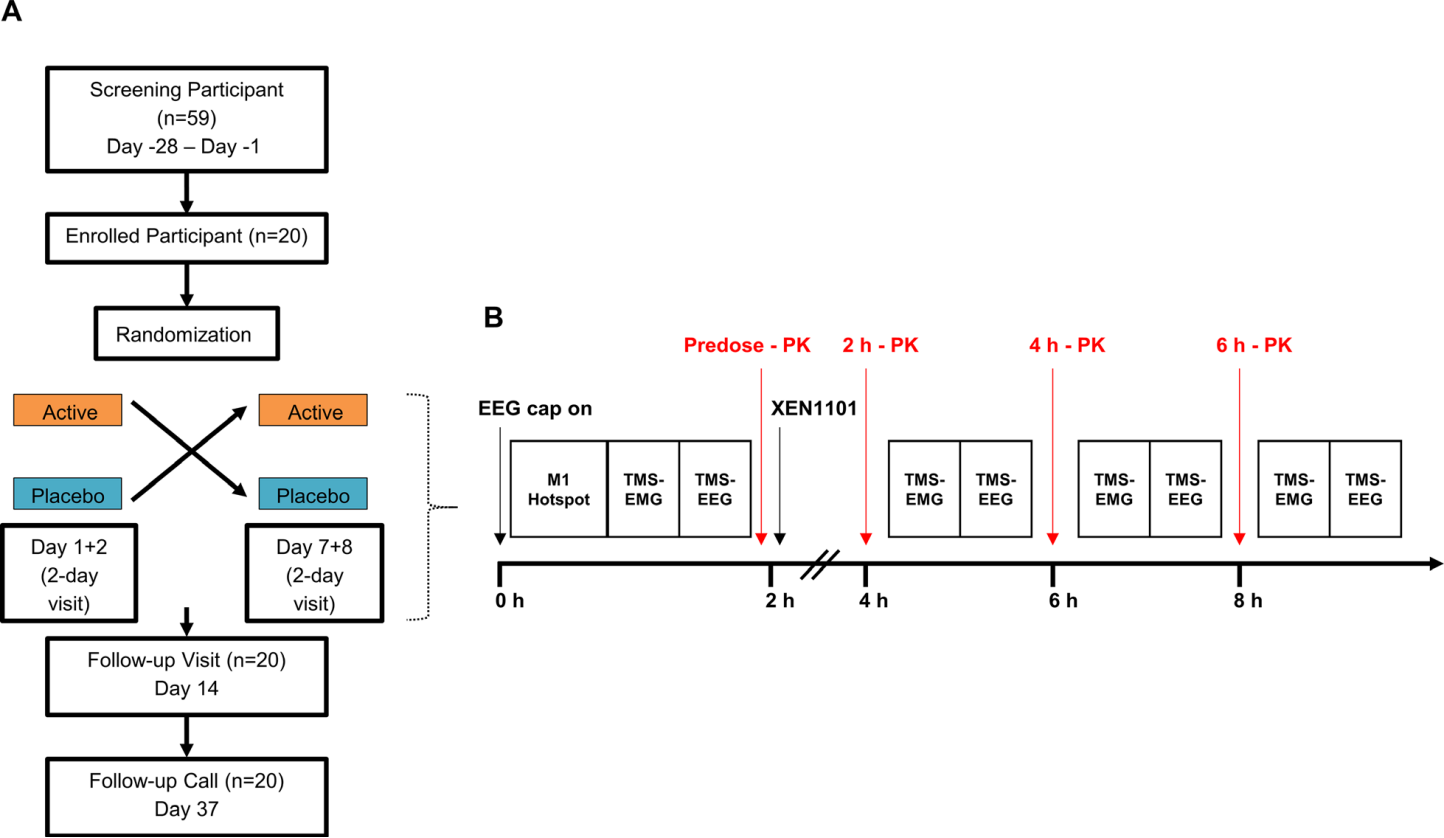
(A) CONSORT diagram of the randomized, double‐blind, placebo‐controlled, two‐period crossover study from screening to the follow‐up call. (B) TMS‐EMG assessments (RMT, AMT, and SICI) and TMS‐EEG procedures undertaken during Day 1 and Day 7. After placing the EEG cap and after localization of left M1 best stimulation target area (hotspot) TMS‐EMG and TMS‐EEG were obtained for each participant and experimental session, before and 2, 4, and 6 h after drug intake. The blood sample was obtained ~5 min before the postdose TMS measurements.

The study investigated the safety, tolerability, and pharmacodynamic profile of single doses of XEN1101 (20 mg) in 20 healthy male subjects using a randomized, double‐blind, placebo‐controlled, two‐period crossover design. All subjects were right‐handed (Edinburgh Handedness Inventory laterality score ≥ 75%).^14^ Participants remained under supervision until discharge on Day 2. Following a 6‐day washout, subjects returned to the unit on Day 7 to receive the alternate treatment and remained under supervision until discharge on Day 8. Subjects returned on Day 14 for a follow‐up and completed a telephone interview on Day 37 (Fig. 1A).

In treatment periods, safety assessments including vital signs, physical examination, electrocardiograms, clinical laboratory evaluations, and Columbia‐Suicide Severity Rating Scale scores were obtained predose and at selected postdose time points. Pharmacokinetics (PK) were assessed with blood samples collected on Day 1 and 7 (predose) and at selected postdose time points on Days 1‐2 and 7‐8. Pharmacodynamic (PD) assessments, including TMS‐EMG and TMS‐EEG measurements, were conducted on Day 1 and 7 at predose and at 2‐ and 4‐hours post dose (Fig. 1B). After blinded review of the PK profiles indicated slow absorption, additional TMS‐EMG and TMS‐EEG assessments were added at 6 hours postdose in the remaining 16 and 8 subjects, respectively.

### Data acquisition

#### TMS‐EMG

We used a Magstim 200^2^ stimulator connected to a figure‐of‐eight coil (diameter 90 mm) through a BiStim (Magstim Co). Single TMS pulses were applied over the hand area of left motor cortex (M1) to elicit motor evoked potentials (MEPs) in the right first digital interosseous (FDI) muscle, recorded using surface EMG (20 Hz to 2 kHz; D360 amplifier, Digitimer, Hertfordshire, UK). Resting motor threshold (RMT), active motor threshold (AMT), and short‐interval intracortical inhibition (SICI) were measured. RMT was determined using the relative frequency method.^15^, ^16^ AMT was recorded while subjects squeezed a manometer at 20% of maximum contraction. SICI was measured using stimuli pairs at interstimulus interval of 2 msec, with 15 conditioning stimulus at 80% AMT and 15 test stimulus 120% RMT.^17^ The position of the FDI hotspot was marked with a felt tip pen on the EEG cap to ensure constant coil placement throughout an experimental session. Furthermore, coil position and orientation relative to the marked position were carefully monitored by the experimenter throughout stimulation and corrected if necessary (i.e., if the participant moved). Importantly, the double‐blind design ensured that no systematic error could be introduced.

#### TMS‐EEG

Subjects were seated with eyes open while fixating. During stimulation subjects listened to a colored masking noise^18^ whose intensity was adjusted individually in each experimental session, until the participant reported that they were unable to hear the TMS‐click*.* TMS‐compatible EEG equipment (BrainAmp MRplus, BrainProducts GmbH) with a 64‐electrode cap (Multitrodes, BrainCap‐Fast'n Easy) was used. 150 consecutive TMS stimuli, with inter‐trial interval of approximately 4 sec (random variation of 20%), were applied at 100% RMT. RMT was obtained before and at 2, 4, and 6 h after drug administration. In each TMS‐EEG session the stimulation intensity was kept relative to the predrug RMT. PK blood samples were obtained ~5 min before each postdose TMS measurement period.

### Data analysis and statistics

#### TMS‐EMG

EMG data were analyzed via Spike2 software (Cambridge Electronic Design). Peak‐to‐peak MEP amplitudes were measured for each trial and averaged per condition. SICI was calculated as the ratio of mean conditioned MEP to mean unconditioned MEP.

Drug‐induced changes in RMT, AMT, and SICI were evaluated using a two‐way rmANOVA (Bonferroni corrected) with a within subject design and a main effect of Time [four levels for RMT and AMT (pre, 2‐, 4‐, and 6‐h postdrug intake) and three levels for SICI (pre, 2‐ and 4‐h postdrug intake)] and Drug (two levels: active and placebo). Missing values were replaced by the average per experimental condition.

#### TMS‐EEG

The analysis of TMS‐EEG data was performed offline, blinded to drug conditions, using Brain Vision Analyzer, Fieldtrip (version 2016, http://www.fieldtriptoolbox.org) and custom scripts in Matlab 2012b version 7.7 (MathWorks, Natick, Mass).^7^

After excluding trials with prominent eye movements, blinks, and muscle artifacts, EEG data were down‐sampled to 1 kHz, segmented into epochs from 1 sec before and after the TMS pulse. A linear interpolation for ± 10 msec was applied to remove the TMS artifact. An average of 3 (1–5 range) bad channels were removed from the EEG, and the signal was reconstructed by interpolating the surrounding electrode signals. Data were notch filtered (50 Hz) and residual artifacts related to the TMS pulse (e.g., TMS recharging artifact, muscle decay artifact), eye‐blinks, saccades, and muscle movement were removed by Independent Component Analysis (ICA). On average, 17 components (range 4–24) were deleted following the procedures described in a previous report.^19^ Finally, remaining data were baseline‐corrected (from −600 to −50 msec), band‐pass filtered (2–80 Hz) and re‐referenced to the average channel signal.

TEPs were calculated by averaging artifact‐free EEG trials for each drug condition. To smooth the signal a low‐pass filter of 45 Hz was applied to TEPs. The average number of trials for each condition is reported in Table 1.

**Table 1:** Number of artefact free EEG trials.

	Number of Artefact Free EEG Trials (Mean, Range)			
	Pre dose	2 h Post	4 h Post	6 h Post
Placebo	128 (90, 142)	121 (98, 136)	125 (105,137)	125 (101,137)
XEN1101	130 (106,149)	124 (102,145)	123 (94,139)	131 (110,141)

Average number and range of artefact free TMS‐EEG trials before and after placebo and XEN1101 intake.

We studied the following TEP components (value in parenthesis denotes the time of interest, TOI): N15‐P25 (15–35 msec), N45 (35–70 msec), P70 (70–80 msec), N100 (80–145 msec), and P180 (145–230 msec). TOIs were chosen on the basis of the grand‐averaged TEPs and kept identical across all analyses. To analyse drug‐induced modulation of TEPs, we selected a region of interest (ROI) that was composed of 27 channels around the stimulation site and the corresponding contralateral site (FC1, FC3, FC5, C1, C3, C5, CP1, CP3, CP5, P1, P3, P5, Cz, CPz, Pz, FC2, FC4, FC6, C2, C4, C6, CP2, CP4, CP6, P2, P4, P6). We analyzed TEP components at four time points (predose, 2, 4, and 6 h post dose, where available) in each of the two sessions (placebo, active drug) in each individual.

To investigate the impact of XEN1101 on TEPs, we applied multiple paired t‐tests (post vs. predrug intake) on the level of individual electrodes within ROI, within each drug condition and each TOI separately. A nonparametric, cluster‐based permutation analysis as implemented in Fieldtrip was used to control for multiple comparisons across channels and time points within TOIs.^20^ Clusters were defined as adjacent time point‐channel pairs for which the t‐statistic exceeded a threshold of *P* < 0.05. Cluster‐level statistics were calculated based on the sum of t‐values within each cluster. Monte Carlo P‐values were computed based on 1500 random permutations and a value of *P* < 0.05 was used as the cluster‐statistical significance threshold for all comparisons.

We applied Spearman correlation analyses to investigate significant correlation between drug‐induced modulation (post minus predrug) of TMS‐EMG/EEG measures and peak plasma concentration of XEN1101.

Finally, to exclude the possibility that changes in TEPs are explained by drug induced changes in RMT reflected by amplitude of MEPs, the change in the percentage of suprathreshold MEPs (>0.5 mV) between pre and postconditions was calculated and used as a covariate in a post hoc multivariate ANOVA with within‐subjects factors CONDITION (predrug, postdrug), and TEP‐PEAK (each significant TEP effect).

## Results

Subjects (*n* = 20) had a mean age ± standard deviation (SD) of 26.6 ± 5.9 years (range 19–40 years), and a mean weight ± SD of 72.3 ± 9.5 kg. They all participated in all requisite study visits and there were no dropouts. One subject did not receive any of the TMS measurements at the 2 h time point in the XEN1101 treatment period due to an adverse event (vomiting). TMS‐EEG procedures could not be completed for another subject at 2 h in the placebo treatment period due to technical problems, so at 2 h this subject only underwent RMT.

### XEN1101 safety, tolerability, and pharmacokinetics

The drug was overall safe and well tolerated. AEs occurring in two or more subjects during XEN1101 treatment included dizziness, somnolence, fatigue, disturbance in attention, and headache (Table 2). Compared to placebo, the only related AE reaching a statistically significant difference (*P* < 0.05) in terms of incidence was dizziness. There were no deaths, SAEs, or withdrawals due to AEs. There were no clinically significant changes in clinical laboratory evaluations, vital signs, or ECG.

**Table 2:** List of adverse events.

System organ class preferred term	Overall	
	XEN1101 20 mg (N = 20)	Placebo (N = 20)
	n (%) E	n (%) E
Any Related^a^ AE	19 (95.0) 65	7 (35.0) 10
Nervous system disorders	17 (85.0) 48	7 (35.0) 8
Dizziness	10 (50.0) 12	1 (5.0) 1
Somnolence	8 (40.0) 11	5 (25.0) 5
Headache	3 (15.0) 6	0
Disturbance in attention	5 (25.0) 5	1 (5.0) 1
Tension headache	4 (20.0) 4	1 (5.0) 1
Ataxia	2 (10.0) 2	0
Diplopia	2 (10.0) 2	0
Vision blurred	2 (10.0) 2	0
General disorders and administration site conditions	7 (35.0) 7	1 (5.0) 1
Fatigue	6 (30.0) 6	1 (5.0) 1
Gastrointestinal disorders	2 (10.0) 3	0
Nausea	2 (10.0) 2	0
Cardiac disorders	2 (10.0) 2	0
Sinus tachycardia	2 (10.0) 2	0

% ‐ (*n*/*N*)*100, *N* for overall column is the total number of subjects in both periods who received XEN1101 20 mg or placebo.

*N*, Number of subjects having a treatment emergent adverse event; *N*, Number of subjects at risk; E, Number of events.

a Related AE: possibly related or definitely related AE.

**Figure 2:**
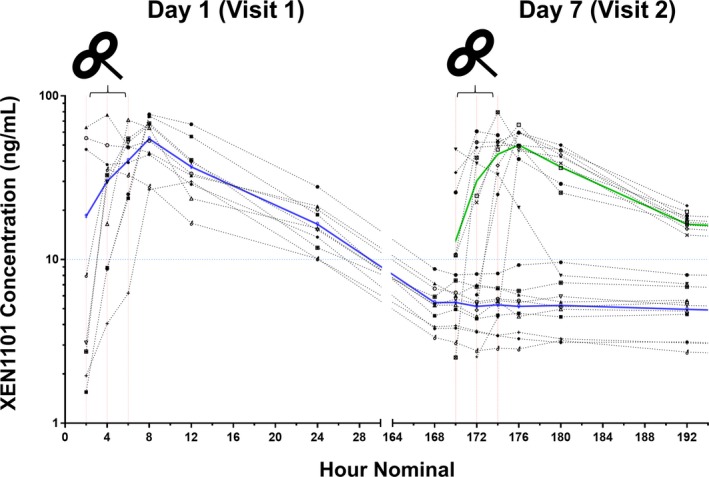
XEN1101 plasma concentrations (ng/mL) are reported as a function of time (hour) for Day 1 (blue line) and Day 7 (green line). XEN1101 shows a profile characterized by a prolonged absorption and XEN1101 was detectable (<8.22 ng/mL) during the placebo period at the second visit (Day7; blue line from 168 to 192 h). TMS assessments, indicated by the black TMS figure‐of‐eight coil, were performed prior to dose and in the ascending part of the absorption curves at 2 h (15.7 ± 21.5) ng/mL, 4 (h 30.2 ± 21.1 ng/mL), and 6 h (44.4 ± 20.2 ng/mL).

The mean ± SD of maximum plasma concentration (C_max_) of XEN1101 was 59.2 ± 13.8 ng/mL (*N* = 20). The median time to peak plasma concentration (T_max_) was 7.8 h (range 1.9 −12 h). The time to peak was slightly earlier in Period 1 compared to Period 2, however, the median XEN1101 T_max_ occured after the TMS measurement time points in both periods. At the TMS assessment time points of 2, 4, and 6 h postdose, mean (±SD) XEN1101 plasma concentrations were 15.7 ± 21.5 ng/mL, 30.2 ± 21.9 ng/mL, and 42.1 ± 19.1 ng/mL, respectively (Fig. 2).

Due to the slow drug elimination, participants who received XEN1101 in Period 1 had small carry‐over drug levels in their placebo arm (highest XEN1101 concentration at start of second period: 8.2 ng/mL). In addition, four participants did not reach XEN1101 concentrations higher than the carry‐over observed in the placebo arm (8.2 ng/mL).

Given the PK profile, XEN1101‐induced modulation of TMS measurements was evaluated as an effect of concentration (using the postdose measure taken during highest drug exposure vs. baseline) and of time (comparisons of 2, 4, and 6 h postdose vs. predose). To remove the potential carry‐over effect, analyses were performed for selected participants (*n* = 16) who showed drug plasma levels higher than the highest concentration detected as a carry‐over in the placebo arm.

### Effects of XEN1101 on TMS‐EMG measures

**Figure 3:**
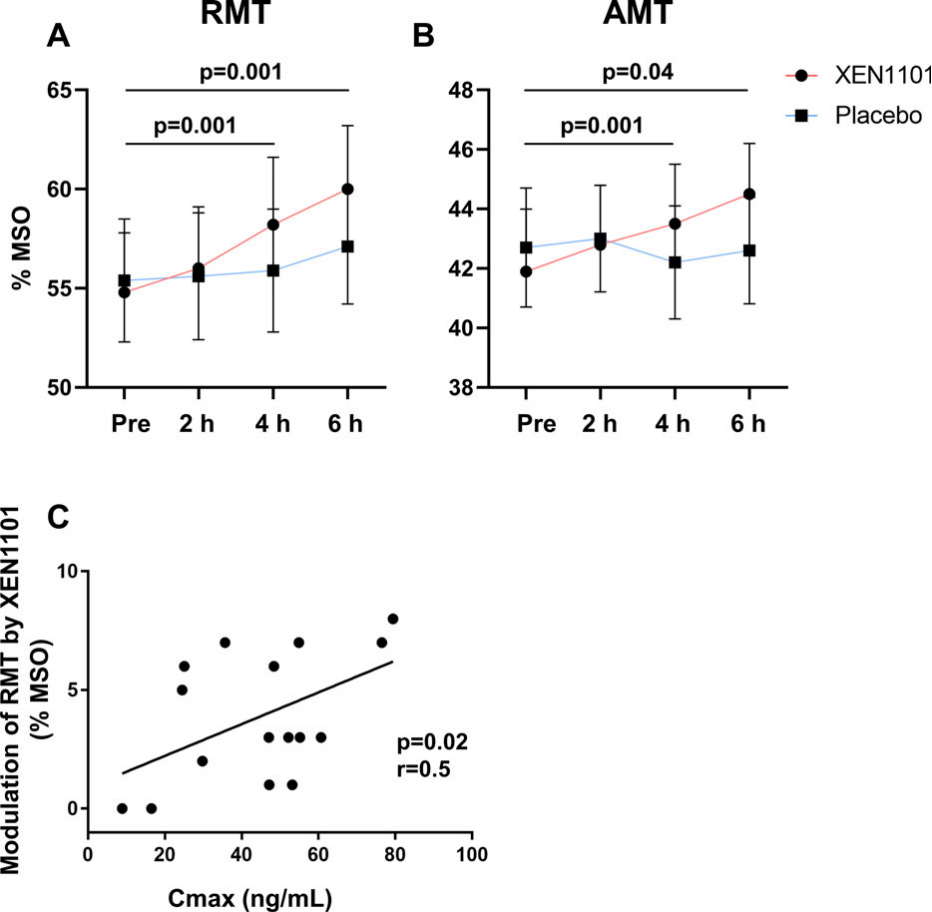
RMT (panel A) and AMT (panel B) mean values ± SEM (%MSO) are reported for each time point (Pre, 2 h, 4 h, and 6 h post drug intake) for XEN1101 (red line) and placebo (blue line). Black lines with *P* values indicate significant effects with respect to baseline. Panel C shows the significant positive correlation between RMT changes (postdrug *minus* predrug observed at the maximum systemic exposure) and XEN1101 plasma levels (ng/mL).

The repeated‐measure ANOVA for RMT showed a significant effect of Drug (*F*_1,15_ = 7.3; *P* = 0.009), Time (*F*_1.3,20_ = 8.1; *P* = 0.006) and a significant interaction between Drug and Time (*F*_3,45_ = 9.7; *P* < 0.001). The interaction was explained by a significant time‐dependent increase in RMT at 4 h (*P* = 0.001) and 6 h (*P* = 0.001) after XEN1101 intake. There were no significant effects for placebo over time (*P* > 0.05). Finally, compared to placebo the RMT was significantly higher for XEN1101 at 4 h (*P* = 0.009) and at 6 h (*P* < 0.001) (Fig. 3A).

The repeated‐measure ANOVA for AMT showed a significant Drug by Time interaction (*F*_1.7, 26_ = 6.4, *P* = 0.007). Compared to predrug state the AMT was significantly increased at 4 h (*P* = 0.001) and at 6 h (*P* = 0.04). In the comparison with placebo, AMT was significantly higher at 4 h (*P* = 0.03) and at 6 h (*P* = 0.001) (Fig. 3B).

XEN1101 did not show significant modulation of SICI (*P* > 0.05). At the time of individual highest XEN1101 concentration, during the RMT assessments (i.e., at 2, 4, or 6 h), there was a significant positive correlation between changes in RMT and Xen plasma concentration (*r* = 0.5; *P* = 0.02; Fig. 3C). Other correlations were not significant (*P* > 0.05).

### Effects of XEN1101 on TEPs

**Figure 4:**
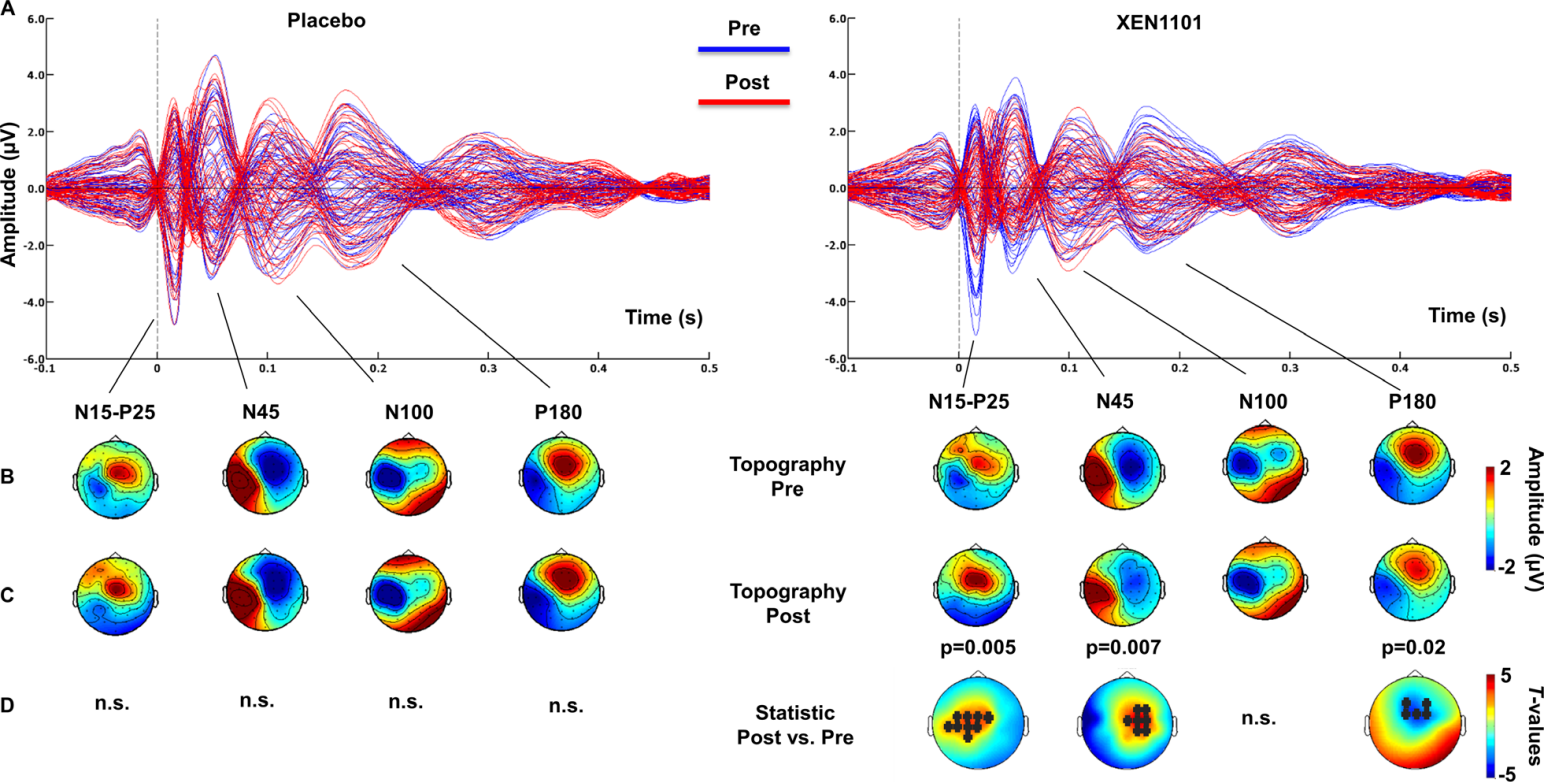
Spatiotemporal profile of TEPs after Placebo and XEN1101 administration. Panel A shows grand‐average (*n* = 16) butterfly plots before (Pre; blue) and after (Post; red) intake of placebo (left) and XEN1101 (right). Each line represents TEPs recorded at a single EEG channel. Topographical scalp distributions of the amplitude (µV) of the main TEP components (N15‐P25, N45, N100, and P180) before and after drug intake are shown in panel B and C respectively. Panel D represents t‐statistic maps of the TEP amplitude showing postdrug versus pre‐drug differences. Nonsignificant results are indicated by n.s. and black asterisks indicate channels that showed a significant drug‐induced modulation.

The spatiotemporal profile of the TMS‐evoked EEG potentials is in line with previous reports^21^ (Fig. 4A and 4). The comparison between predrug conditions (preplacebo versus pre‐XEN1101) did not show significant differences (*P* > 0.05).

#### Concentration analysis

**Figure 5:**
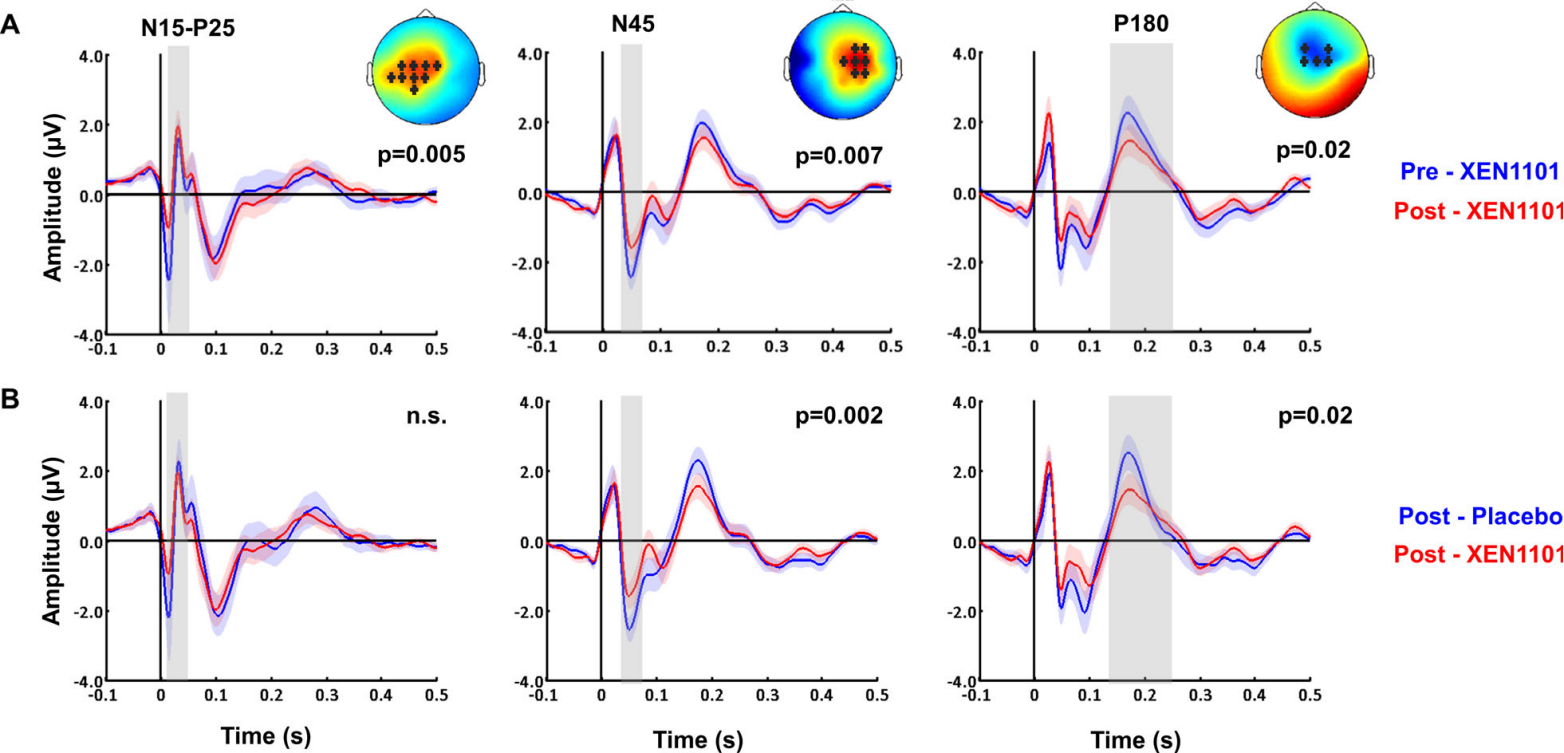
XEN1101 modulation of TEP amplitude at highest concentration compared to pre intake (A) and to time‐matched placebo (B). TEPs grand‐averaged over channels which showed significant drug‐induced effects, indicated by asterisks in the topography plots of *t*‐values. These are TEP data averaged over 16 participants with postdose conditions selected at highest drug exposure during TMS evaluation. Compared to pre‐dose (A, blue) and placebo (B, blue), XEN1101 (red) induced a suppression of the N15‐P25, N45, and P180 components.

During TMS measurement sessions with highest concentration in each individual, compared to baseline, XEN1101 decreased the amplitude of the early TEPs components measured from 15 to 25 msec over the stimulated left motor area (N15‐P25, *P* = 0.005), and at 45 msec (N45, *P* = 0.007) and 180 msec (P180, *P* = 0.02) over contralateral sites (Fig. 5A). Compared to time‐matched placebo, XEN1101 decreased the N45 potential in the contralateral hemisphere (*P* = 0.002), and the P180 amplitude (*P* = 0.02) over fronto‐central sites (Fig. 5B). Drug‐induced changes in TEPs did not correlate with XEN1101 peak plasma levels (*P* > 0.05).

#### Time analysis

**Figure 6:**
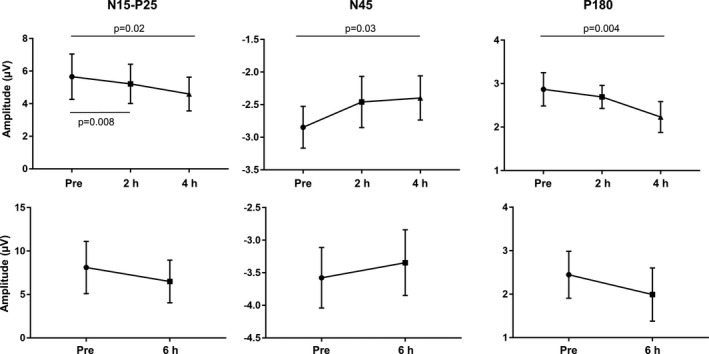
XEN1101 modulation of TEPs amplitude at 2, 4, and 6 h after dosing. Mean amplitude values ± SEM recorded at the level of significant channels before, 2 h (*n* = 15), 4 h (*n* = 16), and 6 h (*n* = 7) after XEN1101 intake. XEN1101 fingerprints which include the reduction in the N15‐P25, N45, and P180 components reflect increasing plasma exposure over time.

The cluster‐based permutation analysis was applied between postdose and predose conditions to test the effects of XEN1101 at 2 h (*n* = 15), 4 h (*n* = 16), and 6 h (*n* = 7) after dosing in subjects with adequate XEN1101 exposure during the first 6 h. Compared to predose, the first N15‐P25 complex was decreased at 2 h (*P* = 0.008) and 4 h (*P* = 0.02). Furthermore, at 4 h after dosing XEN1101 significantly suppressed the N45 (*P* = 0.03), the N100 (*P* = 0.04), and the P180 (*P* = 0.004) (Fig. 6). Other comparisons were not statistically significant (*P* > 0.05).

### Interaction between TEPs and EMG recording

Average MEP amplitudes and the change in the percentage of suprathreshold MEPs (>0.5 mV) between pre and postdrug measurements are calculated and are shown in Table 3. The repeated measures ANOVA showed that there is no interaction between CONDITION and change in the percentage of suprathreshold MEPs, and no three‐way interaction between CONDITION, TEP‐PEAK (N15‐P25, N45, and P180) and change in the percentage of suprathreshold MEPs (all *P* > 0.05).

**Table 3:** List of TMS‐EMG parameters.

	Pre dose			2 h Post			4 h Post			6 h Post		
	RMT	Amplitude	Supra	RMT	Amplitude	Supra	RMT	Amplitude	Supra	RMT	Amplitude	Supra
	%MSO	mV	%	%MSO	mV	%	%MSO	mV	%	%MSO	mV	%
XEN1101	53.65 ± 11.05	0.23 ± 0.14	10.17 ± 12.90	54.74 ± 11.79	0.18 ± 0.13	5.73 ± 9.69	56.75 ± 12.51	0.16 ± 0.09	5.54 ± 7.45	59.06 ± 13.28	0.12 ± 0.07	4.48 ± 6.32
Placebo	54.15 ± 11.42	0.25 ± 0.14	12.92 ± 17.70	54.50 ± 11.68	0.30 ± 0.18	12.86 ± 15.37	54.85 ± 11.72	0.32 ± 0.13	17.88 ± 18.17	56.19 ± 12.20	0.24 ± 0.15	13.67 ± 21.49

Table showing for each measurement point grand averages ± SD of RMT (%MSO), MEPs (mV) and the percentage of suprathreshold MEPs (%).

## Discussion

TMS measurements were incorporated in a healthy volunteer Phase I clinical trial to provide indirect evidence that the investigational drug crosses the BBB and early readouts of pharmacodynamic effects. XEN1101 showed significant plasma concentration dependent reduction of corticospinal and cortical excitability, as measured with RMT and TEPs, respectively. The modulation of TMS‐EDM is consistent with AEDs with similar modes of action and these results support further development of XEN1101 as an AED.

### The effects of XEN1101 on TMS‐EMG measures

Following administration of XEN1101, we observed that the RMT was increased in a time‐ and plasma concentration‐dependent manner in comparison to baseline and placebo. These results provide evidence for XEN1101’s ability to reduce cortical excitability with a strong PK/PD relationship.

RMT and AMT are typically increased by several AEDs blocking sodium channels, such as lamotrigine^7^, ^22^ and carbamazepine,^23^ or opening potassium channels^9^ (i.e., retigabine). Opening K_V_7.2/7.3 potassium channels plays a crucial role in suppressing neuronal excitability as this shifts the membrane potential toward a more hyperpolarized state.

### The effects of XEN1101 on TMS‐EEG measures

Administration of XEN1101 produced statistically significant modulations of TEPs consistent with effects of other AEDs.^7^, ^8^ At the time of the highest plasma levels during TMS assessments XEN1101 decreased the amplitudes of the N15‐P25 complex, the N45, and the P180 potentials. In addition, drug‐induced TEP modulation correlated with drug plasma exposure with strong effects at 4 h after drug intake.

The N15 component is generated in the ipsilateral premotor cortex. The origin of P25 is less clear, but it may reflect activity around ipsilateral motor cortex, ipsilateral cingulate gyrus or supplementary motor area, and in the contralateral cortex.^24^ In a study of patients with progressive myoclonic epilepsy, the P25 waveform was increased, suggesting an association with elevated cortical excitability.^25^ The N15‐P25 complex has been correlated with MEP amplitude, thus providing information about the excitability of the stimulated area.^24^ Following this interpretation and similar to findings with carbamazepine, the reduction of the peak‐to‐peak amplitude of these early components may reflect the drug‐induced reduction of cortical excitability.^8^ In addition, this study is accumulating evidence for another footprint of AED activity, which is the late TMS component (P180). Prior studies have suggested that the P180 may be a residual of the auditory evoked signal.^26^, ^27^ However, given that we applied a masking noise that minimizes the AE,^18^, ^28^ and the prior evidence that drugs like carbamazepine do not modulate the amplitude of the auditory evoked response,^29^ we believe that the XEN1101 effects on the P180 reflect a modulation of the TMS‐evoked brain responses. Finally, XEN1101 suppressed the N45 amplitude which has been linked to GABA‐A receptor mediated neurotransmission by studies that manipulated TEPs with benzodiazepines as GABAergic positive modulators.^30^, ^31^ The N45 reduction could reflect less inhibition, speculatively due to decreased GABA release into the synaptic cleft after hyperpolarization of the presynaptic terminal. Alternatively, the TMS response may not have propagated to contralateral sites given the overall increase in cortical inhibition, which may have reduced the N45 amplitude over distant sites. However, the N45 modulation sits in contrast to the increase produced by lamotrigine and levetiracetam over channels ipsilateral to the stimulated site. This specific action has been speculated to be related to indirect activity of the two AEDs at the level of GABA‐A receptors.^32^, ^33^, ^34^

### Implications and limitations

This study provides compelling evidence that TMS can play a valuable role in providing pharmacodynamic measures of target engagement in early stage trials. Furthermore, a systemically administered drug, such as XEN1101, with central PD effects, such as modulation of TMS outputs, indirectly indicates that the drug crosses the BBB. The introduction of TMS‐EDM in early trials may help avoid excessive exposure of volunteers to high and toxic dosages, thereby limiting AEs, may contribute valuable information about the dose range to be tested in later‐stage trials, and strongly supports the advancement of the new therapeutic into phase 2 clinical development. In addition, the intrinsic neuronal membrane properties and level of cortical excitation and inhibition are relevant mechanisms in epileptogenesis.^35^ Therefore, from a clinical perspective, TMS endpoints may play a valuable role when determining the therapeutic effects of XEN1101 in epilepsy patients.

When considering that TMS‐EEG is approaching clinical translation,^4^, ^36^ it must be taken into account that the TMS pulse can induce unwanted somatosensory responses that have an impact on the cortical signal detected with EEG.^37^ An online TEP quality control during data collection will be crucial to obtain high‐quality and genuine TMS‐evoked brain responses. Furthermore, if protocol allows it, blocks of adjusted and unadjusted stimulation intensity should be assessed under a TMS navigated setting. This would help to understand whether TEP changes reflect an effect of the drug on the generators of TEPs, rather than just reflecting the consequences of a reduction of corticospinal excitability and relative change in RMT.

In addition, it must be noted that given the novel drug pharmacokinetic profile, in some participants TMS measures were not performed at the highest drug exposure. Therefore, our results may be an underestimate of the true pharmacological effect. Furthermore, in some subjects dosed with active drug at the first session, there was measurable XEN1101 in the plasma a week later at the placebo session, although there was no difference in baseline between periods.

In conclusion, this study showed that TMS can provide a landscape of early electro‐diagnostic readouts to help shape future development of new therapeutics. In addition to epilepsy, this approach could be applied in the context of a variety of neurological and neuropsychiatric conditions.

## Conflict of Interest

This research has been funded by Xenon Pharmaceuticals Inc.
